# Clinical Association of Pan-Immune-Inflammation Value with MEFV Mutation Burden and Amyloidosis in Adults with Familial Mediterranean Fever: A Retrospective Cohort Study

**DOI:** 10.3390/jcm15135058

**Published:** 2026-06-29

**Authors:** Ozgur Yilmaz, Osman Erinc, Ozan Cemal Icacan, Gulseren Goktolga Erkoca, Recep Demirci, Sengul Aydin Yoldemir, Murat Akarsu

**Affiliations:** 1 Department of Internal Medicine, Kanuni Sultan Suleyman Training and Research Hospital, Istanbul 34303, Turkey; 2 Department of Rheumatology, Kanuni Sultan Suleyman Training and Research Hospital, Istanbul 34303, Turkey; 3 Department of Medical Genetics, Kanuni Sultan Suleyman Training and Research Hospital, Istanbul 34303, Turkey; 4 Department of Nephrology, Kanuni Sultan Suleyman Training and Research Hospital, Istanbul 34303, Turkey

**Keywords:** familial mediterranean fever (FMF), pan-immune-inflammation value (PIV), MEFV mutation, amyloidosis

## Abstract

**Background and Objectives:** Familial Mediterranean fever (FMF) is a hereditary autoinflammatory disease characterized by recurrent inflammatory attacks and a risk of AA amyloidosis. Although inflammatory activity may persist during attack-free periods, reliable biomarkers of subclinical inflammation remain limited. The pan-immune-inflammation value (PIV), a composite index derived from circulating immune cell counts, has emerged as a marker of systemic inflammation. This study investigated the association between PIV, MEFV mutation burden, and amyloidosis in patients with FMF during attack-free periods. **Materials and Methods:** This retrospective cross-sectional study included 386 adult patients with FMF followed at a tertiary rheumatology clinic. Patients were stratified by MEFV mutation status into three groups: Group 1 (genetically non-confirmatory FMF or low-penetrance/non-causative MEFV variants such as E148Q), Group 2 (single pathogenic mutation), and Group 3 (biallelic pathogenic mutations). Patients were also categorized by amyloidosis status. PIV was calculated as (neutrophil count × platelet count × monocyte count)/lymphocyte count using complete blood count parameters obtained during attack-free visits. Associations between PIV and clinical characteristics were evaluated using correlation and logistic regression analyses, and discriminative performance was assessed using receiver operating characteristic (ROC) curve analysis. Statistical significance was set at *p* < 0.05. **Results:** PIV levels differed significantly across genotype-defined groups (median: 172, 329.3, and 479.5 in Groups 1–3, respectively; *p* < 0.001) and were higher in patients with amyloidosis than in those without amyloidosis (540.5 vs. 218.1; *p* < 0.001). In multivariable logistic regression analysis, PIV remained independently associated with both biallelic pathogenic mutation status (OR = 1.007, 95% CI: 1.003–1.010, *p* < 0.001) and the presence of amyloidosis (OR = 1.002, 95% CI: 1.001–1.003, *p* < 0.001). ROC analysis showed an AUC of 0.853 for distinguishing Group 3 from Group 1 (cut-off 337; sensitivity 80.7%, specificity 77.0%) and an AUC of 0.814 for discriminating patients with and without amyloidosis (cut-off 316.4; sensitivity 86.0%, specificity 65.6%). **Conclusions:** PIV was independently associated with MEFV mutation burden and amyloidosis in patients with FMF during attack-free periods. These findings suggest that PIV may reflect the inflammatory burden associated with genetic mutation load and amyloidosis in FMF. Prospective longitudinal studies are warranted to externally validate these findings and further clarify the relationship between PIV, inflammatory burden, and disease severity in FMF.

## 1. Introduction

Familial Mediterranean fever (FMF) is the most common hereditary autoinflammatory disorder, characterized by recurrent episodes of fever and serositis. It predominantly affects populations originating from the Mediterranean basin, particularly Turkish, Armenian, Arab, and Jewish communities, a distribution largely attributed to genetic predisposition and historical founder effects [1,2]. FMF results from mutations in the MEFV gene, which encodes pyrin, a key regulator of inflammasome activation and interleukin-1β-mediated inflammatory responses [3,4].

The M694V mutation is one of the pathogenic variants associated with a more severe disease course and earlier onset. Importantly, this mutation has also been associated with colchicine resistance and a dramatically elevated incidence of secondary AA amyloidosis, a critical driver of long-term morbidity and mortality in FMF. On the other hand, variations such as E148Q have varying penetrance and an inconsistent relationship with disease manifestations; therefore, their clinical importance remains controversial [5,6,7,8]. Because the pathogenic significance of E148Q remains controversial, careful interpretation of this variant is warranted in genotype–phenotype analyses.

Although FMF attacks are typically short-lived, accumulating evidence indicates that inflammatory activity may persist even during clinically quiescent periods. This subclinical inflammation is believed to contribute to progressive organ injury and long-term complications, particularly AA amyloidosis [9,10,11]. Conventional acute-phase reactants such as C-reactive protein (CRP), erythrocyte sedimentation rate (ESR), and serum amyloid A (SAA) are widely used to monitor inflammatory activity in FMF; however, their dynamic fluctuations may limit their ability to detect low-grade chronic inflammation or accurately reflect cumulative inflammatory burden [10,12].

Consequently, increasing attention has been directed toward hematological indices derived from routine complete blood counts. Among these, the neutrophil-to-lymphocyte ratio (NLR) and platelet-to-lymphocyte ratio (PLR) have been investigated as surrogate indicators of inflammatory activity. However, available studies have reported inconsistent diagnostic and prognostic performances of these indices in FMF, suggesting that they may not consistently capture both overt and subclinical inflammation [11,13,14,15]. These limitations have prompted interest in more integrative inflammatory indices, among which the Pan-Immune-Inflammation Value (PIV) has recently emerged as a promising candidate across several clinical settings [16,17,18,19]. Recent studies have further demonstrated that composite immune-inflammatory biomarkers derived from multiple hematologic parameters may provide a more comprehensive assessment of systemic inflammatory burden and disease-related inflammatory activity across different chronic disorders [20,21].

Recently, PIV has been proposed as a composite inflammatory index that depicts the balance between inflammatory activation and immune regulation. With the inclusion of circulating neutrophil, monocyte, platelet, and lymphocyte counts, PIV provides a multidimensional estimate of the systemic inflammatory status. First described in the field of oncology, increased PIV levels have been associated with tumor progression and worse survival [16,17,22]. Subsequent studies have reported similar associations with adverse clinical outcomes in cardiovascular and immune-mediated diseases, suggesting that PIV may represent a broadly accessible marker of systemic inflammation across different pathological contexts [18,19,23,24]. Given the central role of persistent inflammation in FMF, PIV may therefore represent a biologically plausible indicator of inflammatory burden even during attack-free periods.

Although PIV has recently been evaluated in FMF, available evidence has mainly focused on overall disease severity and amyloidosis [25]. Its relationship with MEFV mutation burden and genotype-based stratification, however, remains insufficiently characterized. Considering that MEFV mutations are key determinants of clinical severity and amyloidosis development in FMF [9,10,11,13], evaluating inflammatory indices such as PIV across genotype-defined subgroups may provide additional insight into subclinical inflammatory activity and disease stratification beyond conventional clinical severity scores.

Therefore, the present study aimed to investigate whether PIV differs across MEFV genotype-defined subgroups in patients with FMF and to evaluate its association with inflammatory and clinically relevant disease characteristics, including the presence of AA amyloidosis. In addition, the study explored the relationship of PIV with routinely used inflammatory markers and its potential complementary role in reflecting inflammatory burden during attack-free periods.

## 2. Materials and Methods

### 2.1. Study Design and Settings

This single-center retrospective cross-sectional study was conducted in the adult rheumatology outpatient clinic of a tertiary-care hospital. Consecutive patients aged ≥18 years with a diagnosis of FMF according to the Tel-Hashomer criteria [26] and followed between 1 January 2022, and 30 June 2025 were included. Clinical, demographic, and laboratory data were retrieved from institutional electronic medical records, and MEFV mutation results were obtained from routine genetic reports. All data were anonymized prior to analysis. The study was reported in accordance with the Strengthening the Reporting of Observational Studies in Epidemiology guidelines [27].

### 2.2. Study Population

A total of 418 adult patients with FMF were identified from rheumatology outpatient clinic records. After applying the predefined exclusion criteria, 386 patients with complete demographic, clinical, laboratory, and MEFV genotyping data were included in the final analysis. Patients were stratified into three groups according to MEFV allelic status. Group 1 consisted of patients with genetically non-confirmatory FMF or low-penetrance/non-causative MEFV variants, including variants of uncertain clinical significance such as E148Q. These patients fulfilled established Tel-Hashomer diagnostic criteria and demonstrated clinically compatible FMF phenotypes as assessed by experienced rheumatologists regardless of MEFV mutation status. Group 2 included monoallelic carriers with a single heterozygous pathogenic MEFV mutation (e.g., M694V, V726A, M680I, or M694I). Group 3 comprised patients with biallelic pathogenic genotypes, either homozygous (e.g., M694V/M694V) or compound heterozygous (e.g., M694V/V726A or M680I/M694V). Disease severity was assessed using the International Severity Scoring System for FMF (ISSF) [28]. Baseline demographic and clinical characteristics were systematically obtained from the medical records, including age, sex, age at disease onset, disease duration, and family history of FMF. Amyloidosis was defined based on histopathological confirmation of AA amyloid deposition in tissue biopsy specimens recorded in the medical files. All patients were receiving colchicine therapy as part of routine clinical management during the study period; however, detailed treatment-related variables, including colchicine dose, treatment adherence, treatment duration, colchicine resistance or intolerance status, and recent attack frequency, were not consistently documented in the medical records because of the retrospective study design.

**Figure 1:**
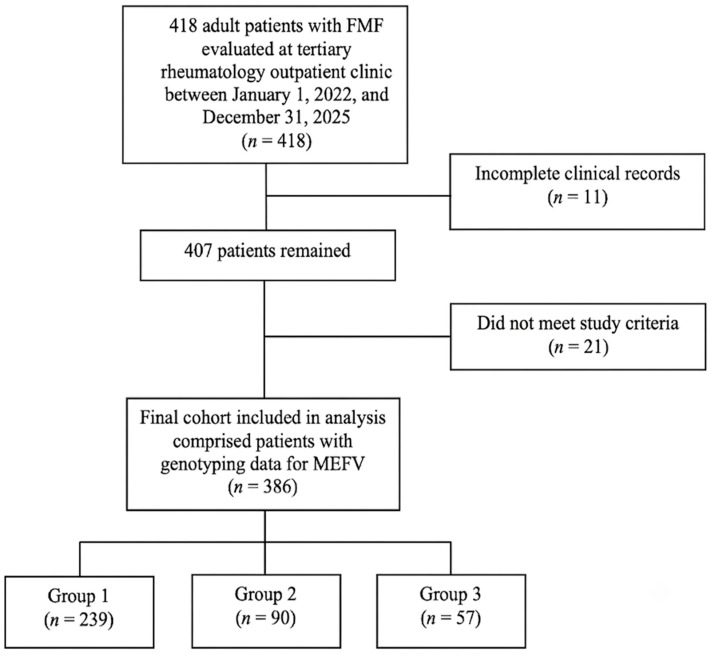
Flowchart of patient selection.

Patients were excluded if they were younger than 18 years, pregnant or breastfeeding, or had active malignancy, chronic liver disease, severe or decompensated heart failure, or advanced chronic kidney disease requiring dialysis or associated with unstable clinical status. Additional exclusion criteria included coexisting systemic autoimmune or rheumatologic diseases (e.g., rheumatoid arthritis or systemic lupus erythematosus), acute infections or other clinically significant inflammatory conditions, and treatment with long-term immunosuppressive or biologic agents, including interleukin-1 inhibitors. Patients with evidence of acute infection identified through retrospective review of clinical examination records and accompanying laboratory findings documented in the electronic medical database were excluded. Patients with incomplete medical records, missing essential laboratory parameters, or unavailable MEFV genotyping required for genotype classification were also excluded. The patient selection process is shown in Figure 1.

The primary outcome of the study was the association between PIV and MEFV mutation allele burden in patients with FMF. Secondary outcomes included the associations of PIV with amyloidosis and with relevant clinical and laboratory parameters.

### 2.3. Data Collection and Laboratory Measurements

Disease severity and laboratory measurements were evaluated during attack-free periods, defined as at least two weeks after complete resolution of FMF symptoms. However, the exact duration of the attack-free interval beyond this minimum two-week period was not consistently documented in the medical records because of the retrospective study design. Venous blood and morning spot urine samples were collected after a minimum of 8 h of fasting during routine outpatient assessments. Complete blood count parameters, serum CRP, and serum creatinine levels were measured in the same accredited central laboratory using standardized automated methods. The estimated glomerular filtration rate (eGFR) was calculated using the CKD-EPI creatinine equation [29].

### 2.4. Genetic Analysis and PIV Calculation

Mutational analysis of the MEFV gene was performed using PCR-based assays followed by reverse hybridization strip testing. Commercially available kits (FMF StripAssay^®^, ViennaLab Diagnostics, Vienna, Austria) were used according to the manufacturer’s instructions. The panel screened for the most common FMF-associated variants, including M694V, M680I, V726A, and E148Q, as well as additional variants included in the commercial assay. Genetic results were retrospectively obtained from medical records. Comprehensive MEFV sequencing or extended auto inflammatory disease gene panel testing was not uniformly available for all patients because of the retrospective design and variability in routine clinical genetic evaluation. Although some patients underwent additional genetic investigations during clinical follow-up, these data were not consistently accessible in the medical records and therefore could not be systematically incorporated into the analyses. Accordingly, genotype classification was standardized according to the routinely available targeted MEFV panel results.

The pan-immune-inflammation value (PIV) was calculated using complete blood count parameters according to the formula: PIV = (neutrophil × platelet × monocyte)/lymphocyte, expressed as absolute cell counts (×10^9^/L). To minimize temporal mismatch, only measurements obtained during attack-free visits and closest to the ISSF assessment were considered for analysis.

### 2.5. Statistical Analysis

Statistical analyses were performed using SPSS Statistics for Windows, version 26.0 (IBM Corp., Armonk, NY, USA). As this was a retrospective cross-sectional study, no a priori sample size calculation was performed, and all eligible patients meeting the inclusion criteria during the study period were included in the analysis. The distribution of continuous variables was assessed using skewness and kurtosis values (±1.5). Normally distributed variables were presented as mean ± standard deviation (SD) with minimum–maximum values, whereas non-normally distributed variables were expressed as median and interquartile range (Q1–Q3). Categorical variables were summarized as frequencies and percentages.

Comparisons among the three genotype groups were performed using one-way analysis of variance (ANOVA) for normally distributed variables and the Kruskal–Wallis H test for non-normally distributed variables. When the Kruskal–Wallis test was significant, post hoc pairwise comparisons were conducted using Bonferroni-adjusted tests. Comparisons between two independent groups were performed using the Mann–Whitney U test for continuous variables. Categorical variables were compared using the Pearson chi-square test or Fisher’s exact test when appropriate.

Correlations between PIV and clinical or laboratory parameters were assessed using Spearman’s rank correlation analysis. Factors associated with genetic mutation status and amyloidosis were evaluated using univariate and multivariable logistic regression analyses. Univariate analyses were performed using separate logistic regression models for each variable. In the multivariable logistic regression model, all clinically relevant variables were entered simultaneously (enter method), regardless of their significance in univariate analysis, to account for potential confounding effects. Results were reported as regression coefficients (B), standard errors (SE), odds ratios (OR), and 95% confidence intervals (CI). Model calibration was assessed using the Hosmer–Lemeshow goodness-of-fit test, and multicollinearity was evaluated using tolerance and variance inflation factor (VIF) values. Additionally, sensitivity analyses excluding patients with amyloidosis were performed to further evaluate whether the observed associations persisted independently of amyloidosis-related inflammation. Because severe FMF phenotype may overlap with amyloidosis, renal dysfunction, and persistent inflammatory activity, an additional multivariable logistic regression model incorporating clinically relevant variables including disease duration, CRP, eGFR, and amyloidosis status was constructed to further assess the independent association of PIV with biallelic pathogenic mutation status. The discriminatory performance of PIV for differentiating genetic groups and for discriminating amyloidosis status was evaluated using receiver operating characteristic (ROC) curve analysis. Additionally, comparative ROC analyses including CRP, ESR, NLR, and PLR were performed to assess the discriminative performance of inflammatory biomarkers for differentiating Group 1 and Group 3. Optimal cut-off values were determined according to the Youden index, and the corresponding area under the curve (AUC), sensitivity, specificity, positive predictive value (PPV), and negative predictive value (NPV) were calculated. Correlated ROC curve comparisons were performed using the DeLong method, and Holm correction was applied for multiple pairwise comparisons. A *p* value < 0.05 was considered statistically significant in all analyses.

## 3. Results

A total of 386 patients with FMF were included in the final analysis. The mean age of the cohort was 36.62 ± 11.24 years, and the mean age at FMF diagnosis was 21.99 ± 7.01 years. Of the participants, 263 (68.1%) were female. Amyloidosis was identified in 43 patients (11.1%), and a positive family history of FMF was present in 176 patients (45.6%).

Regarding MEFV mutations, 156 patients (40.4%) were classified as genetically non-confirmatory FMF, whereas heterozygous M694V was the most frequently detected pathogenic variant. Detailed distributions of MEFV genotypes and genotype-based subgroup classifications are presented in Table 1.

Age at diagnosis differed significantly among the groups and was lowest in Group 3 compared with Groups 1 and 2 (*p* < 0.001). A positive family history of FMF was more frequent in Groups 2 and 3 than in Group 1 (*p* < 0.001). The prevalence of amyloidosis increased markedly across the groups, from 0.4% in Group 1 to 10.0% in Group 2 and 57.9% in Group 3 (*p* < 0.001). In addition, WBC, neutrophil, and monocyte counts, as well as CRP and ESR levels, were higher in Group 3, whereas lymphocyte counts and eGFR values were lower. Serum creatinine levels were also higher in Group 3 (all *p* < 0.05). Similarly, NLR and PLR levels also differed significantly across the genotype-defined groups (both *p* < 0.001) (Table 2).

**Figure 2:**
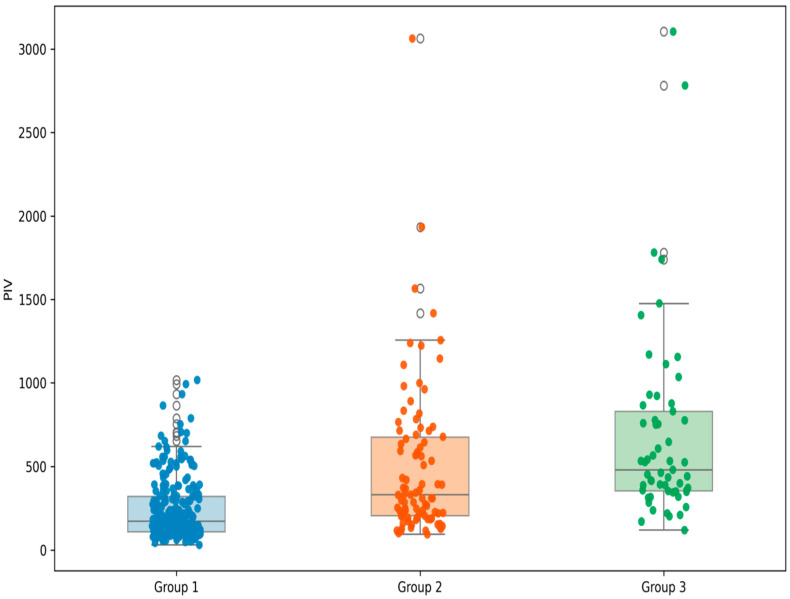
PIV levels according to MEFV genotype groups. Group 1: genetically non-confirmatory FMF or low-penetrance/non-causative MEFV variants such as E148Q (*n* = 239); Group 2: single pathogenic MEFV mutation (heterozygous) (*n* = 90); Group 3: biallelic pathogenic MEFV mutations (homozygous or compound heterozygous) (*n* = 57). Boxes represent the interquartile range (IQR) with the median indicated by the horizontal line; whiskers indicate the data range, and dots represent individual patient values.

PIV levels differed significantly across the three genotype groups. Median PIV levels increased from Group 1 to Group 3 (172, 329.3, and 479.5, respectively; *p* < 0.001). Pairwise comparisons showed that PIV levels were higher in Group 2 than in Group 1 (*p* < 0.001), whereas Group 3 had the highest levels and differed significantly from both Group 1 (*p* < 0.001) and Group 2 (*p* = 0.002) (Figure 2).

An additional sensitivity analysis was performed after exclusion of patients with amyloidosis. After exclusion of amyloidosis cases, 238 patients in Group 1, 81 in Group 2, and 24 in Group 3 were analyzed, and PIV levels remained significantly different across the groups (*p* < 0.001). Post hoc analyses demonstrated significantly higher PIV levels in Groups 2 and 3 compared with Group 1 (both *p* < 0.001), whereas the difference between Groups 2 and 3 was not statistically significant (*p* = 0.054).

When stratified according to amyloidosis status, patients with amyloidosis exhibited significantly higher PIV levels than those without amyloidosis. A total of 43 patients with amyloidosis and 343 without amyloidosis were evaluated, and median PIV levels were 540.5 and 218.1, respectively (*p* < 0.001).

Spearman correlation analyses demonstrated significant associations between PIV and several inflammatory and hematologic parameters across genotype-defined groups. In Group 1, PIV was positively correlated with MCHC (ρ = 0.282, *p* < 0.001) and negatively correlated with MPV (ρ = −0.221, *p* = 0.001). In Group 2, PIV showed positive correlations with MPV (ρ = 0.210, *p* = 0.047) and CRP (ρ = 0.293, *p* = 0.005), whereas inverse correlations were observed with hemoglobin (ρ = −0.297, *p* = 0.004) and basophil count (ρ = −0.391, *p* < 0.001). In Group 3, PIV demonstrated positive correlations with basophil count (ρ = 0.294, *p* = 0.026) and CRP (ρ = 0.290, *p* = 0.029), while negative correlations were observed with eosinophil count (ρ = −0.350, *p* = 0.008) and eGFR (ρ = −0.326, *p* = 0.013).

When patients were stratified according to amyloidosis status, several significant correlations with PIV were observed. In patients without amyloidosis, PIV was positively correlated with ISSF score (ρ = 0.345, *p* < 0.001), MCH (ρ = 0.138, *p* = 0.011), MCHC (ρ = 0.226, *p* < 0.001), eGFR (ρ = 0.109, *p* = 0.043), CRP (ρ = 0.301, *p* < 0.001), and ESR (ρ = 0.211, *p* < 0.001), and negatively correlated with age at diagnosis (ρ = −0.131, *p* = 0.015) and basophil count (ρ = −0.132, *p* = 0.014). In patients with amyloidosis, PIV was negatively correlated with eGFR (ρ = −0.388, *p* = 0.010).

Logistic regression analysis was performed to identify factors associated with Group 3 compared with Group 1 (Model 1). In the univariate analysis, CRP and PIV were significantly associated with Group 3. In the multivariable model, both CRP and PIV remained independently associated with Group 3 status (PIV: OR = 1.007, 95% CI: 1.003–1.010, *p* < 0.001) (Table 3).

An additional logistic regression analysis was performed to identify factors associated with Group 3 compared with Group 1 (Model 2). In the multivariable model, higher age at diagnosis remained associated with lower odds of Group 3 status, whereas disease duration, CRP, and PIV remained independently associated with Group 3 status. PIV remained significantly associated with Group 3 status after adjustment for disease duration, amyloidosis, eGFR, and CRP (OR = 1.006, 95% CI: 1.003–1.010, *p* < 0.001) (Table 4).

Logistic regression analysis was performed to identify factors associated with the presence of amyloidosis. In the univariate analysis, both PIV and CRP were significantly associated with amyloidosis. In the multivariable model, PIV remained independently associated with the presence of amyloidosis (OR = 1.002, 95% CI: 1.001–1.003, *p* < 0.001), together with CRP (Table 5).

**Figure 3:**
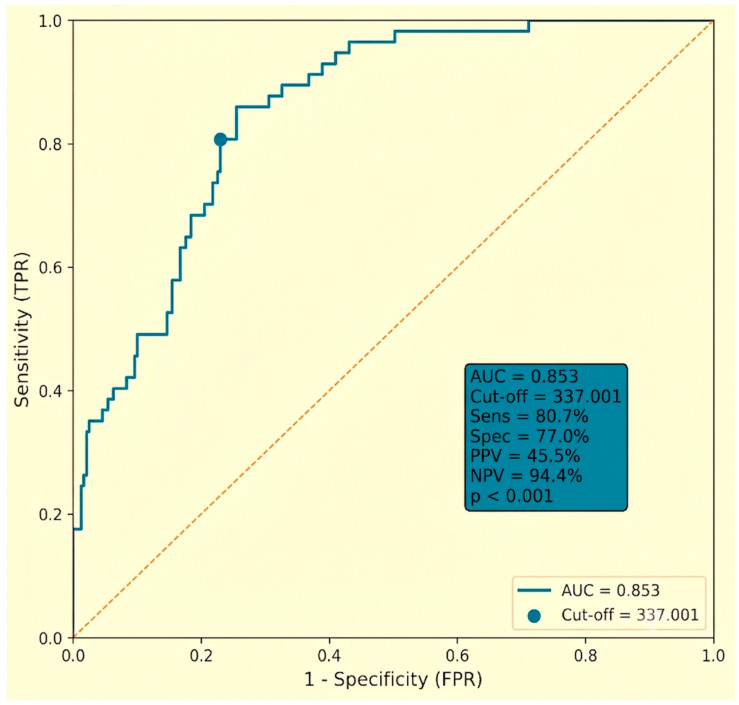
ROC Curve of PIV for Discriminating Group 1 FMF from Group 3 FMF.

ROC analysis demonstrated an AUC of 0.853 for distinguishing Group 3 from Group 1. The optimal cut-off value determined using the Youden index was 337, with a sensitivity of 80.7%, a specificity of 77.0%, a PPV of 45.5%, and an NPV of 94.4% (Figure 3). When Group 2 and Group 3 were compared, the AUC was 0.652 (*p* = 0.001), with an optimal cut-off value of 311.9, sensitivity of 86.0%, specificity of 48.9%, PPV of 51.6%, and NPV of 84.6%.

**Figure 4:**
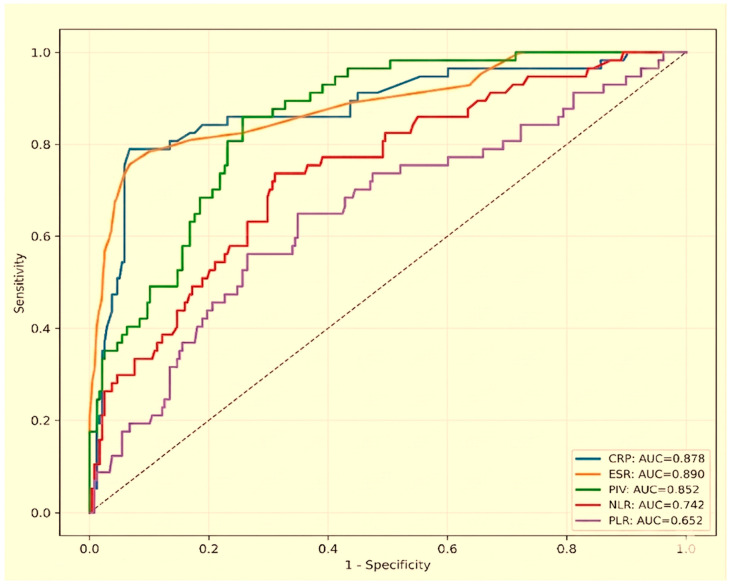
Comparative ROC Analysis Results of PIV, CRP, ESR, NLR, and PLR for Discriminating Group 3 Disease from Group 1.

Additional comparative ROC analyses were performed to compare the discriminative performance of PIV with conventional inflammatory markers, including CRP, ESR, NLR, and PLR, for differentiating Group 1 and Group 3. PIV demonstrated good discriminative performance and remained comparable to CRP and ESR in pairwise ROC curve comparisons according to DeLong analyses with Holm correction. In contrast, PIV demonstrated significantly higher discriminatory performance than both NLR and PLR. Similarly, CRP and ESR each showed significantly greater discriminative performance compared with NLR and PLR (Table 6 and Table 7 and Figure 4).

**Figure 5:**
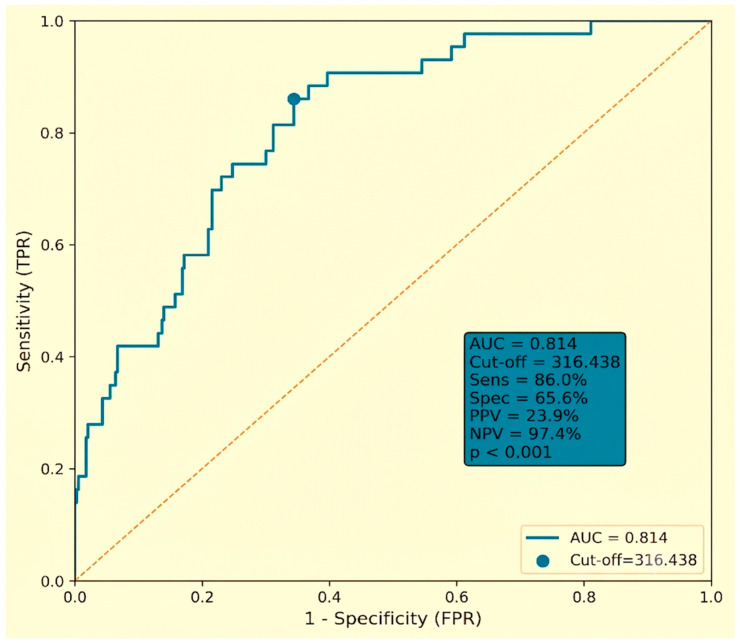
ROC curve showing the discriminatory performance of PIV for identifying patients with amyloidosis.

ROC analysis evaluating the discriminatory performance of PIV for identifying patients with amyloidosis yielded an AUC of 0.814 (*p* < 0.001). The optimal cut-off value was 316.4, corresponding to a sensitivity of 86.0% and a specificity of 65.6%, with a PPV of 23.9% and an NPV of 97.4% (Figure 5).

## 4. Discussion

In the present study, PIV levels showed a gradual increase among the MEFV mutation-defined groups and were highest among biallelic pathogenic variant carriers. These results may suggest an association between a greater mutation burden and more profound systemic inflammatory activity in FMF. Notably, the stepwise increase in PIV across genotype-defined groups suggests that this composite index may capture gradations in inflammatory intensity linked to mutation load. Although the higher prevalence of amyloidosis in Group 3 may contribute to the observed increase in PIV levels, significant differences in PIV across genotype-defined groups persisted after exclusion of patients with amyloidosis. However, because patients with biallelic pathogenic mutations also exhibited substantially higher rates of amyloidosis, greater inflammatory activity, and more pronounced renal involvement, residual confounding cannot be completely excluded. Therefore, the observed association may partly reflect the overall inflammatory burden associated with more severe FMF phenotypes rather than mutation burden alone. In addition, higher PIV levels were accompanied by increased neutrophil and monocyte counts together with lower lymphocyte levels, which may reflect enhanced innate inflammatory activation in patients with higher mutation burden. Nevertheless, given the cross-sectional design of the study, these findings should be interpreted cautiously and do not imply causality.

Another important finding was the significantly higher PIV levels observed in patients with amyloidosis. AA amyloidosis is one of the most severe long-term complications of FMF and has been associated with persistent subclinical inflammation even during attack-free periods [9,10]. Consistent with our findings, Ocak et al. also reported higher PIV levels in patients with FMF-related amyloidosis [25]. In our cohort, the higher prevalence of amyloidosis among patients with biallelic pathogenic mutations may further support the relationship between greater mutation burden and sustained inflammatory activity.

Previous studies have shown that M694V-dominant MEFV mutations, particularly homozygous or compound heterozygous exon 10 variants, are associated with an increased risk of AA amyloidosis in FMF [30,31]. Similarly, Bektas et al. reported a high prevalence of exon 10 mutations, especially p.M694V, in a large Turkish AA amyloidosis cohort [32]. In addition, Gershoni-Baruch et al. and Mukhin et al. highlighted that modifier genes, including SAA1, may also contribute to amyloidosis susceptibility in FMF [33,34].

Moreover, PIV showed significant discriminating performance in the detection of amyloidosis patients. Lower PIV levels may be more useful for exclusion than for confirmation of inflammatory involvement associated with amyloidosis, given the comparatively low positive predictive value and high negative predictive value. The relatively low prevalence of amyloidosis in the study population may be partially responsible for this pattern. Overall, these findings suggest that PIV may reflect inflammatory activity associated with amyloidosis in FMF.

In addition to the group-based comparisons, PIV remained independently associated with the presence of amyloidosis after adjustment for age, sex, and CRP. This observation suggests that the relationship between PIV and amyloidosis is not fully explained by the conventional inflammatory burden reflected by CRP. This difference may be related to the composite nature of PIV, which integrates multiple immune cell lineages rather than reflecting a single acute-phase reactant. Although the per-unit effect appears modest, the wide range of PIVs suggests that higher levels may still correspond to clinically meaningful differences in inflammatory burden. Nevertheless, the relatively small effect sizes indicate that the magnitude of these associations should be interpreted with caution. However, causal relationships cannot be established, and this association may suggest that PIV could reflect a broader inflammatory profile observed in patients with amyloidosis in FMF.

Patients with biallelic pathogenic MEFV mutations were diagnosed at a younger age and exhibited a more pronounced inflammatory laboratory profile compared with other genotype groups. These findings are consistent with previous genotype–phenotype studies showing that exon 10-dominant and biallelic mutations are associated with earlier disease onset and more severe inflammatory expression in FMF [4,6,7]. Moradian et al. and Marek-Yagel et al. similarly reported that heterozygous individuals may also develop clinical disease, although with more heterogeneous and generally milder phenotypes than patients carrying biallelic pathogenic variants [35,36]. In parallel with this inflammatory profile, NLR and PLR levels also increased significantly across genotype-defined groups.

It should also be acknowledged that Group 1 comprised both genetically non-confirmatory FMF patients and carriers of low-penetrance variants such as E148Q, resulting in a relatively heterogeneous reference category. Although this classification enabled stratification according to overall mutation burden, alternative grouping approaches might have yielded different effect estimates. Therefore, the potential influence of group composition on the observed between-group differences should be considered when interpreting the findings.

Spearman correlation analyses demonstrated significant associations between PIV and several inflammatory parameters across genotype-defined groups. Similar correlations between PIV and inflammatory markers have also been reported in other inflammatory and immune-mediated conditions [16,19,23].

PIV was also positively correlated with ISSF disease severity score and acute-phase reactants, including CRP and ESR, particularly in patients without amyloidosis. In patients with amyloidosis, PIV showed a negative correlation with eGFR, suggesting a possible association between inflammatory activity and renal involvement in this subgroup. These findings are consistent with previous studies demonstrating persistent subclinical inflammation in FMF even during attack-free periods [37]. However, although laboratory assessments were performed at least two weeks after symptom resolution, inflammatory markers may normalize at different rates among patients. Therefore, residual post-attack inflammatory activity may have contributed, at least in part, to the observed variability in PIV and other inflammatory parameters.

Another noteworthy finding was that, even after multivariable adjustment, including CRP, amyloidosis status, disease duration, and eGFR, higher PIVs continued to be independently associated with biallelic pathogenic mutation status. These results imply that the observed association may not be entirely explained by traditional inflammatory markers or severe disease-related organ involvement alone. It should be emphasized that genotype status represents an inherent genetic characteristic that precedes biomarker measurement. Accordingly, the logistic regression analyses were used to evaluate whether the associations between PIV and genotype-defined disease categories remained independent of potential confounders. Therefore, these findings should be interpreted as evidence of cross-sectional association rather than biological causation. In this respect, the present findings may extend previous genotype–phenotype studies by suggesting that a composite hematologic index could provide complementary information regarding inflammatory burden in FMF [4,7]. Similar observations have also been reported in studies demonstrating that PIV reflects systemic inflammatory activity across different clinical settings [16,17,18,19]. However, treatment-related and attack-related factors, including colchicine dose, treatment adherence, colchicine resistance or intolerance, biologic therapy exposure, and attack frequency, may influence both inflammatory activity and hematological inflammatory markers. Because these variables were not consistently available, their potential contribution to the observed associations could not be fully assessed.

ROC analyses further supported the clinical relevance of PIV. Comparative ROC analyses demonstrated that the discriminative performance of PIV remained comparable to CRP and ESR while exceeding that of NLR and PLR. These findings suggest that PIV may capture inflammatory information similar to conventional acute-phase reactants while also providing broader hematologic inflammatory profiling through integration of multiple immune cell populations. However, the absence of a significant difference between PIV and CRP/ESR indicates that PIV should not be interpreted as a superior inflammatory marker but rather as a complementary composite inflammatory index in FMF. Accordingly, the potential clinical utility of PIV may lie in providing an integrated assessment of the immune-inflammatory response through a single CBC-derived parameter rather than in replacing established biomarkers such as CRP and ESR. PIV also showed discriminatory performance for differentiating patients with biallelic pathogenic mutations from those without pathogenic mutations, whereas its performance was more modest for distinguishing monoallelic from biallelic carriers. This observation may reflect the more heterogeneous inflammatory phenotypes reported in heterozygous patients [35,36]. However, the ROC-derived cut-off values identified in the present study should be considered exploratory rather than definitive clinical thresholds because of the retrospective cross-sectional design and lack of external validation.

### Study Limitations

Several limitations should be considered when interpreting the findings of this study. The retrospective cross-sectional design precludes causal inference between PIV levels and disease outcomes. In addition, the single-center setting may limit the generalizability of the findings to broader FMF populations. Although the cohort size was relatively large, the number of patients with certain clinical outcomes, particularly amyloidosis, remained limited and may have affected the statistical power of subgroup analyses. Inflammatory markers were evaluated only during attack-free periods, and potential fluctuations during acute attacks were not assessed. Furthermore, because detailed treatment-related and attack-related clinical data were not consistently available, the potential influence of treatment control, residual disease activity, and subclinical inflammatory burden on PIV levels could not be fully evaluated. Additionally, detailed information regarding colchicine resistance status, severe inflammatory manifestations, and long-term clinical outcomes was not consistently documented because of the retrospective study design. Patients receiving biologic therapies, including interleukin-1 inhibitors, were excluded from the study; therefore, the generalizability of the findings to FMF patients treated with contemporary biologic agents such as canakinumab may be limited. In addition, the classification of variants such as E148Q remains controversial and may have influenced subgroup assignment in a subset of patients. Another important limitation is that genetic analysis was based on a targeted PCR-based MEFV mutation panel rather than comprehensive MEFV sequencing or extended autoinflammatory disease gene testing. Because of the retrospective design and variability in routine clinical genetic evaluation, broader genetic investigations were not uniformly available for all patients. Therefore, the possibility of undetected rare MEFV variants cannot be completely excluded, which may have affected genotype classification in a small number of patients. In addition, or alternative hereditary autoinflammatory syndromes cannot be completely excluded. In addition, some patients in Group 1 were clinically diagnosed and treated as FMF despite non-confirmatory genetic findings, which may have influenced the clinical and genetic homogeneity of this subgroup. Finally, the absence of longitudinal follow-up prevented assessment of whether elevated PIV levels precede future complications. Additionally, the ROC-derived thresholds identified in this study require validation in independent prospective cohorts before potential clinical application. Similarly, the comparative discriminatory performance analyses of inflammatory biomarkers require external validation in independent cohorts.

## 5. Conclusions

In conclusion, PIV levels were higher in patients with greater MEFV mutation burden and in those with amyloidosis during attack-free periods. These findings suggest that PIV may reflect a more pronounced inflammatory phenotype in FMF. Because PIV is derived from routinely available hematological parameters, it may represent a simple, inexpensive, and readily available measure of disease-related inflammatory burden in FMF. However, these findings should be interpreted in light of unavailable treatment-related and attack-related clinical variables that may have influenced inflammatory activity and PIV levels. Prospective multicenter studies with longitudinal follow-up are required to further validate these findings and clarify its potential role in reflecting inflammatory burden and disease severity in FMF.

## Figures and Tables

**Table 1:** Distribution of MEFV Genetic Mutations in Patients with FMF.

Genetic Mutation, *n* (%)	(*n* = 386)
Genetically non-confirmatory FMF	156 (40.4)
M694V HTZ	70 (18.1)
E148Q HTZ	32 (8.3)
M680I HTZ	20 (5.2)
V726A HTZ	16 (4.1)
M694V HTZ, V726A HTZ	9 (2.3)
M694V HMZ	10 (2.6)
R761H HTZ	8 (2.1)
E148Q HTZ, M694V HTZ	7 (1.8)
M694V HTZ, M680I HTZ	6 (1.6)
E148Q HTZ, M680I HTZ	6 (1.6)
A744S HTZ	5 (1.3)
E148Q HMZ	5 (1.3)
Other rare mutations (<1%)	36 (9.3)

HTZ, heterozygous; HMZ, homozygous. Categorical variables were presented as number (percentage). The detailed genotype distribution shown in this table represents raw MEFV mutation frequencies. Genotype-defined study groups used in subsequent analyses were categorized separately according to the presumed pathogenic significance and allelic burden of detected variants.

**Table 2:** Comparison of demographic, clinical and laboratory characteristics according to MEFV mutation allele status in patients with FMF.

Parameters	Group 1 (*n* = 239)	Group 2 (*n* = 90)	Group 3 (*n* = 57)	*p*
Age, years	36.28 ± 11.09	37.58 ± 11.15	36.51 ± 12.10	0.646 ^f^
Gender, Female/Male, *n* (%)	171 (71.5)/68 (28.5)	60 (66.7)/30 (33.3)	32 (56.1)/25 (43.9)	0.074 ^†^
Age at diagnosis, years	23 (19–31)	20.5 (16–23)	15 (13–17)	<0.001 ^c^
Disease duration, years	10 (5–16)	16 (11–23)	19 (12–28)	<0.001 ^g^
Family history of FMF, *n* (%)	72 (30.3)	56 (62.2)	48 (84.2)	<0.001 ^†^
ISSF score	3 (2–4)	7 (6–8)	9 (8–10)	<0.001 ^a^
Amyloidosis, *n* (%)	1 (0.4)	9 (10)	33 (57.9)	<0.001 ^‡^
Leukocyte count, 10^3^/µL	6.80 (5.56–8.43)	6.60 (5.78–8.04)	8.12 (6.26–9.34)	0.011 ^b^
Red blood cell count, 10^6^/µL	4.5 (4.3–4.9)	4.6 (4.4–5)	4.61 (4.11–4.91)	0.746 ^¶^
Hemoglobin, g/dL	13.11 ± 1.43	12.93 ± 1.45	13.20 ± 1.56	0.512 ^f^
Hematocrit, %	40.2 ± 4.06	40.75 ± 4.34	40.09 ± 4.91	0.525 ^f^
Platelet count, 10^3^/µL	270 (230–307)	278.5 (222–360)	287 (224–345)	0.230 ^¶^
MCH, pg	28.3 (27.5–29.3)	28.9 (27–29.4)	28.9 (27.5–29.2)	0.577 ^¶^
MCHC, g/dL	32.7 (32.3–33.8)	33 (32.7–33.7)	33.2 (32.5–33.8)	0.132 ^¶^
MPV, fL	10.4 (9.40–10.6)	10.5 (9.6–11.4)	10.1 (9.4–11.4)	0.244 ^¶^
Neutrophil count, 10^3^/µL	3.62 (2.87–4.99)	4.14 (3.58–5.15)	5.25 (3.9–6.85)	<0.001 ^a^
Lymphocyte count, 10^3^/µL	2.3 (2–2.8)	2 (1.50–2.60)	1.9 (1.4–2.4)	<0.001 ^e^
Monocyte count, 10^3^/µL	0.41 (0.32–0.53)	0.57 (0.44–0.78)	0.78 (0.59–0.89)	<0.001 ^a^
Eosinophil count, 10^3^/µL	0.17 (0.08–0.19)	0.14 (0.09–0.20)	0.13 (0.05–0.18)	0.148 ^¶^
Basophil count, 10^3^/µL	0.03 (0.02–0.04)	0.02 (0.01–0.04)	0.04 (0.03–0.06)	<0.001 ^b^
Creatinine, mg/dL	0.70 (0.62–0.80)	0.76 (0.65–0.92)	1.19 (0.7–1.54)	<0.001 ^a^
eGFR, mL/min/1.73 m^2^	111.8 (100.7–117.5)	105.7 (94–113)	84.8 (76.9–112.1)	<0.001 ^c^
CRP, mg/L	2 (1–4.3)	12 (2.54–22)	18 (13–31)	<0.001 ^a^
ESR, mm/h	6 (3–10)	15 (8.60–21)	25 (17–36)	<0.001 ^a^
NLR	1.62 (1.21–2.32)	2.42 (1.57–2.95)	2.58 (1.90–4.13)	<0.001 ^g^
PLR	120.36 (96.11–143.18)	131.25 (101–190)	142.14 (118.57–175)	<0.001 ^g^

Group 1 = genetically non-confirmatory FMF or low-penetrance/non-causative MEFV variants such as E148Q; Group 2 = single pathogenic MEFV mutation (heterozygous); Group 3 = biallelic pathogenic MEFV mutations (homozygous or compound heterozygous). MCH, mean corpuscular hemoglobin; MCHC, mean corpuscular hemoglobin concentration; MPV, mean platelet volume; eGFR, estimated glomerular filtration rate; CRP, C-reactive protein; ESR, erythrocyte sedimentation rate; NLR, neutrophil lymphocyte ratio; PLR, platelet–lymphocyte ratio. Data are presented as mean ± standard deviation (SD), median (interquartile range: Q1–Q3), or number (percentage). † = Pearson chi-square test; ‡ = Fisher’s exact test; f = one-way ANOVA; ¶ = Kruskal–Wallis H test. Pairwise comparisons were performed using the Mann–Whitney U test following the Kruskal–Wallis test. a: Group 3 > Group 2 > Group 1; b: Group 3 > Group 1 > Group 2; c: Group 1 > Group 2 > Group 3; e: Group 1 > Group 2 and Group 3; g: Group 2 and Group 3 > Group 1. A *p* value < 0.05 was considered statistically significant.

**Table 3:** Univariate and Multivariable Logistic Regression Analyses of Factors Associated with Group 3 FMF Compared with Group 1 FMF: Model 1.

	Univariate Model					Multivariable Model				
Variables	B	SE	*p*	OR	95% CI	B	SE	*p*	OR	95% CI
Age at diagnosis	−0.375	0.056	<0.001	0.687	0.615–0.767	−0.455	0.096	<0.001	0.634	0.525–0.766
Gender (Female)	−0.675	0.303	0.026	0.509	0.281–0.922	−0.877	0.601	0.145	0.416	0.128–1.352
CRP	0.116	0.017	<0.001	1.123	1.086–1.161	0.086	0.021	<0.001	1.090	1.046–1.136
PIV	0.005	0.001	<0.001	1.005	1.003–1.006	0.007	0.002	<0.001	1.007	1.003–1.010

B, regression coefficient; SE, standard error; OR, odds ratio; CI, confidence interval; CRP, C-reactive protein; PIV, pan-immune-inflammation value. Univariate analyses were performed using separate logistic regression models for each variable. In the multivariable model, all variables were entered simultaneously. Model fit statistics were as follows: χ^2^ = 200.712, *p* < 0.001; Cox & Snell R^2^ = 0.494; Nagelkerke R^2^ = 0.789. No evidence of multicollinearity was observed (all tolerance values > 0.20 and VIF < 5). The Hosmer–Lemeshow test indicated good model calibration (*p* = 0.936). A *p* value < 0.05 was considered statistically significant.

**Table 4:** Univariate and Multivariable Logistic Regression Analyses of Factors Associated with Group 3 FMF Compared with Group 1 FMF: Model 2.

	Univariate Model					Multivariable Model				
Variables	B	SE	*p*	OR	95% CI	B	SE	*p*	OR	95% CI
Gender (Female)	−0.675	0.303	0.026	0.509	0.281–0.922	−0.292	0.666	0.661	0.746	0.202–2.755
Age at diagnosis	−0.375	0.056	<0.001	0.687	0.615–0.767	−0.481	0.114	<0.001	0.618	0.495–0.773
Disease duration	0.081	0.015	<0.001	1.085	1.054–1.117	0.072	0.033	0.030	1.075	1.007–1.148
Amyloidosis	5.791	1.037	<0.001	327.25	42–2499	2.581	1.349	0.056	13.209	0.940–185
eGFR	−0.054	0.009	<0.001	0.947	0.930–0.965	−0.002	0.023	0.930	0.998	0.954–1.044
CRP	0.116	0.017	<0.001	1.123	1.086–1.161	0.066	0.019	0.001	1.068	1.028–1.110
PIV	0.005	0.001	<0.001	1.005	1.003–1.006	0.006	0.002	<0.001	1.006	1.003–1.010

B: regression coefficient; SE: standard error; OR: odds ratio; CI: confidence interval; eGFR: estimated glomerular filtration rate; CRP: C-reactive protein; PIV: pan-immune-inflammation value. The dependent variable was severe clinical course, and the reference category was mild clinical course. In univariate analyses, each variable was evaluated using a separate logistic regression model. In the multivariable model, sex, age at diagnosis, disease duration, amyloidosis, eGFR, CRP, and PIV were entered simultaneously into the model. For categorical variables, the reference categories were male sex and absence of amyloidosis. Multivariable model statistics were as follows: χ^2^ = 209.782; *p* < 0.001. Cox & Snell R^2^ = 0.509; Nagelkerke R^2^ = 0.814. The Hosmer–Lemeshow goodness-of-fit test yielded χ^2^ = 3.043 and *p* = 0.932, indicating adequate model fit. A *p* value < 0.05 was considered statistically significant.

**Table 5:** Univariate and multivariable logistic regression analyses of factors associated with the presence of amyloidosis.

	Univariate Model					Multivariable Model				
Variable	B	SE	*p*	OR	95% CI	B	SE	*p*	OR	95% CI
Age	0.021	0.014	0.120	1.022	0.994–1.050	0.022	0.017	0.200	1.022	0.989–1.056
Gender (Female)	−0.490	0.331	0.138	0.613	0.321–1.171	−0.263	0.397	0.507	0.768	0.353–1.674
CRP	0.071	0.012	<0.001	1.073	1.049–1.099	0.056	0.012	<0.001	1.058	1.033–1.083
PIV	0.003	0.000	<0.001	1.003	1.002–1.004	0.002	0.000	<0.001	1.002	1.001–1.003

B, regression coefficient; SE, standard error; OR, odds ratio; CI, confidence interval; CRP, C-reactive protein; PIV, pan-immune-inflammation value. Univariate analyses were performed using separate logistic regression models for each variable. In the multivariable model, age, gender, CRP, and PIV were entered simultaneously. Model fit statistics were as follows: Omnibus χ^2^ = 78.194, *p* < 0.001; Cox & Snell R^2^ = 0.184; Nagelkerke R^2^ = 0.365. No evidence of multicollinearity was observed (all tolerance values > 0.20 and VIF < 5). The Hosmer–Lemeshow test indicated acceptable model calibration (*p* = 0.082). A *p* value < 0.05 was considered statistically significant.

**Table 6:** ROC Analysis of Inflammatory Parameters for Discriminating Group 1 and Group 3 FMF Patients.

Parameter	AUC	95% CI	*p*	Holm *p*	Cutoff	Sensitivity	Specificity
CRP	0.878	0.816–0.933	<0.001	<0.001	12	0.79	0.93
ESR	0.890	0.827–0.943	<0.001	<0.001	16	0.81	0.95
PIV	0.852	0.799–0.899	<0.001	<0.001	311.9	0.86	0.74
NLR	0.742	0.665–0.812	<0.001	<0.001	2.08	0.74	0.69
PLR	0.652	0.566–0.731	<0.001	<0.001	130.5	0.65	0.65

Optimal cutoff values were determined using the Youden index and represent the specific threshold values for each parameter. AUC, area under curve; CI, confidence interval; CRP, C-reactive protein; ESR, erythrocyte sedimentation rate; PIV, Pan-Immune-Inflammation Value; NLR, neutrophil lymphocyte ratio; PLR, platelet–lymphocyte ratio.

**Table 7:** Pairwise Comparisons of ROC Curve Areas for CRP, ESR, PIV, NLR, and PLR Parameters in Discriminating Between Group 1 and Group 3.

Comparison	AUC 1	AUC 2	AUC Difference	DeLong *p*	Holm *p*
CRP vs. ESR	0.878	0.890	−0.013	0.728	0.908
CRP vs. PIV	0.878	0.852	0.025	0.454	0.908
CRP vs. NLR	0.878	0.742	0.135	<0.001	0.003
CRP vs. PLR	0.878	0.652	0.226	<0.001	<0.001
ESR vs. PIV	0.890	0.852	0.038	0.199	0.598
ESR vs. NLR	0.890	0.742	0.148	<0.001	<0.001
ESR vs. PLR	0.890	0.652	0.239	<0.001	<0.001
PIV vs. NLR	0.852	0.742	0.110	<0.001	<0.001
PIV vs. PLR	0.852	0.652	0.201	<0.001	<0.001
NLR vs. PLR	0.742	0.652	0.091	0.015	0.062

DeLong tests were used for pairwise comparisons of correlated AUC values. A positive AUC difference indicates that the first parameter had a higher AUC value than the second parameter. Holm correction was applied for multiple pairwise comparisons. AUC, area under curve; CRP, C-reactive protein; ESR, erythrocyte sedimentation rate; PIV, pan-immune-inflammation value; NLR, neutrophil lymphocyte ratio; PLR, platelet–lymphocyte ratio.

## Data Availability

The data that support the findings of this study are available from the corresponding author upon reasonable request.

## Abbreviations

FMF	familial Mediterranean fever
PIV	pan-immune-inflammation value
ROC	receiver operating characteristic
CRP	C-reactive protein
ESR	erythrocyte sedimentation rate
NLR	neutrophil-to-lymphocyte ratio
PLR	platelet-to-lymphocyte ratio
ISSF	International Severity Scoring System for FMF
eGFR	estimated glomerular filtration rate
SD	standard deviation
SE	standard error
OR	odds ratio
CI	confidence interval
AUC	area under the curve
PPV	positive predictive value
NPV	negative predictive value
IQR	interquartile range
